# Exploring Binocular Visual Attention by Presenting Rapid Dichoptic and Dioptic Series

**DOI:** 10.3390/brainsci14050518

**Published:** 2024-05-20

**Authors:** Manuel Moreno-Sánchez, Elton H. Matsushima, Jose Antonio Aznar-Casanova

**Affiliations:** 1 Facultad de Psicologia, Department Section of Cognitive Processes, Universitat de Barcelona, 08035 Barcelona, Spain; manelsg@gmail.com; 2 Institut of Neuroscience, Universitat de Barcelona, 08028 Barcelona, Spain; 3 Programa de Pós-Graduação em Medicina (Neurologia and Eurociências), Niterói 24020-140, RJ, Brazil; eh_matsushima@id.uff.br; 4 Institute of Psychology, Universidade Federal Fluminense, Niterói 24020-140, RJ, Brazil

**Keywords:** visual awareness, visuo-spatial attention, binocular vision, attentional blink, dichoptic vision, binocular competition, Rapid Serial Visual Presentation (RSVP)

## Abstract

This study addresses an issue in attentional distribution in a binocular visual system using RSVP tasks under Attentional Blink (AB) experimental protocols. In Experiment 1, we employed dichoptic RSVP to verify whether, under interocular competition, attention may be captured by a monocular channel. Experiment 2 was a control experiment, where a monoptic RSVP assessed by both or only one eye determines whether Experiment 1 monocular condition results were due to an allocation of attention to one eye. Experiment 3 was also a control experiment designed to determine whether Experiment 1 results were due to the effect of interocular competition or to a diminished visual contrast. Results from Experiment 1 revealed that dichoptic presentations caused a delay in the type stage of the Wyble’s eSTST model, postponing the subsequent tokenization process. The delay in monocular conditions may be further explained by a visual attenuation, due to fusion of target and an empty frame. Experiment 2 evidenced the attentional allocation to monocular channels when forced by eye occlusion. Experiment 3 disclosed that monocular performance in Experiment 1 differs significantly from conditions with interocular competition. While both experiments revealed similar performance in monocular conditions, rivalry conditions exhibit lower detection rates, suggesting that competing stimuli was not responsible for Experiment 1 results. These findings highlight the differences between dichoptic and monoptic presentations of stimuli, particularly on the AB effect, which appears attenuated or absent in dichoptic settings. Furthermore, results suggest that monoptic presentation and binocular fusion stages were a necessary condition for the attentional allocation.

## 1. Introduction

Attention is a cognitive mechanism that selects relevant information from perceptual and cognitive systems while withdrawing and attenuating other stimuli [1,2]. Sensory stimuli are more readily detected (and brought into consciousness) and discriminated when attention is directed towards them, leading to faster behavioral responses compared to unattended items (e.g., [3,4]) Attention also influences mental effort, or the cognitive resources needed to sustain mental activities [5].

Prior studies have explored the dissociation between the detection of perceptual targets (both visual and auditory) and the orienting of attention towards those targets (e.g., [6,7,8,9,10,11]). For instance, subjects may fixate on a central visual target while covertly directing attention towards a peripheral target [12]. The present study investigates the attentional distribution through binocular visual system while dissociating stimuli presented to each eye. To the best of our knowledge, research regarding this issue is currently missing.

The dichoptic presentation of rapid serial visual presentation (RSVP) is a case of interocular competition, and binocular rivalry is the most studied phenomenon on interocular competition [13]. In dichoptic RSVP (DRSVP), such interocular competition would only affect attentional selection on the onset of RSVPs, i.e., the first phase of binocular rivalry, known as the onset of rivalry, but not the perceptual alternation that has been estimated to arise at around two seconds [14], beyond the time range of DRSVP.

Two opposing theories tried to account for the experimental evidence. One states an early competition between two eyes (e.g., [15,16]) and the other hypothesized a late competition between visual representations (e.g., [17,18]). Recently, these theories have been fused into a hybrid model, in which rivalry is considered the culmination of a cascade of distributed neuronal events [13]. The perceptual processes that trigger the onset of rivalry and those that promote the perceptual alternation cannot be the same. Therefore, the neuronal circuits that mediate the suppression of the stimulus of one eye could differ from ones promoting the dominance of the stimulus of the other eye. These attentional processes can modulate neuronal activity in multiple stages of the visual processing hierarchy [19,20,21]. Considering these theories, the issue investigated here is determining how does attention operates at the onset of rivalry in DRSVP. Attentional selection would occur in the early division of monocular channels or in a late high-order representation of stimuli.

Attention also varies over time. Temporal constraints in the deployment of visual attention can be assessed using RSVP tasks, where two targets are presented sequentially. Typically, the second target is difficult to detect, resulting in unawareness of its presence when presented shortly after the first target. This phenomenon known as Attentional Blink (AB) can offer valuable insights into the nature of visual awareness, especially when elicited by dichoptic and dioptic RSVPs, shedding light on their temporal dynamics, limitations, neural mechanisms, and the interplay between automatic and controlled processes. The typical detection performance pattern involves a deficit in the detection of the second target during the temporal interval between 200 to 500 ms after the onset of the first target, followed by a recovery of the detection rate of the second target after this interval [22,23]. Another characteristic of the time course of AB effect is the Lag 1 sparing. When the second target appears just after the first target (less than 200 ms), it is more detectable than when second target appears 200 ms after first target. Thus, AB protocols can be aimed at understanding the time course of stimuli awareness in different RSVP.

The Episodic Simultaneous Type-Serial Token Model (eSTST) [24] proposes that visual encoding is geared towards enhancing the episodic structure of information stored in working memory (WM), thereby enforcing the episodic distinction between separately presented targets. In AB experimental protocols, the visual attention system encodes targets (or types, in eSTST) successively presented as a single episode (or token) in WM. However, when distractors occur between two targets, WM encodes two episodes (two separate tokens). eSTST extends the STST model [25] by introducing the concept of a “blaster”, which receives excitatory input from all stimuli and allocates attention to stimuli that meet type criteria or instruction-defined features. Additionally, the blaster receives inhibitory input from the binding pool, which associates the first target (T1) with a token. In cases where successive targets occur, continuous excitatory signals sustain the blaster’s attention on T1. However, when distractors appear after T1, the binding pool sends inhibitory signals to the blaster, disengaging attention to facilitate the tokenization of T1. After approximately 500 ms, the binding pool completes the tokenization process and the blaster (attentional processes) reengages to register T2 [24].

There is evidence suggesting that attention to dichoptically presented concurrent targets in a Rapid Serial Visual Presentation (RSVP) task is not subject to cross-modal restrictions, implying that the source of the AB effect must reside within the specific sensory system [26,27]. Considering the neurophysiological segregation within the visual system, different early visual processes may have varying effects on attentional control. The binocular fusion system is one of the visual processes involved in DRSVP.

A proper fused perception is dependent on sensory fusion, motor fusion, and on the integration of other visual processes, such as accommodative vergence [28] and even in DRSVP. Sensory fusion is the process of combining the images from each eye into a single image and it is dependent on striate and extrastriate areas of the cortex, but mostly on the striate cortex [29]. Motor fusion is the mechanism of fusional vergencial movements, the disjunctive eye movements that keep the eyes aligned on a near target [28]. Accommodative convergence, the automatic change in vergence in response to changes in accommodation [28], which occurs under monocular conditions even on the occluded eye, also plays a role in our experimental settings.

The present study aims to address the gap in the existing experimental evidence regarding attentional distribution in the binocular visual system using RSVP tasks under AB experimental protocols. We investigated whether the attentional mechanism supporting awareness needs a precise coupling of at least three mechanisms of the binocular visual system: vergence accommodation, accommodative vergence, and binocular fusion. When any mismatch occurs, visual processing is affected, subsequently affecting the attentional processes underlying visual awareness, which would be detected in the pattern of targets detection of the AB effect protocols under DRSVP.

In Experiment 1, we employed DRSVP, presenting RSVP separately to each eye [30], either two slightly different RSVP or the same RSVP. The goal was to verify whether under interocular competition, attention on the onset of rivalry was captured by a monocular channel or by a visual representation of stimulus, observing the effects of these experimental settings in AB protocols. Experiment 2 was a control experiment, where a single RSVP (monoptic presentation) was assessed by both eyes or to only one eye while the other was physically occluded. This experiment aimed to determine whether Experiment 1 monocular condition results were due to an allocation of attention to one eye. Experiment 3 was also a control experiment designed to determine whether Experiment 1 results were due to the effect of interocular competition present on DRSVP or to a diminished visual contrast.

## 2. Experiment 1—Dichoptic Presentations

The primary objective of the initial experiment is to investigate the distribution of attentional resources between two slightly different DRSVP series. The physical characteristics of the stimulus, such as visual saliency and contrast in luminance in color, can capture the response of the stimulus in one or another direction. Therefore, attention can strongly influence the dominance of a stimulus in interocular competition [31,32]. Stimuli must be standardized in these photometric aspects, such as the black letters used in our DRSVPs.

In a similar way, attention can increase global contextual characteristics that improve the dominance of a stimulus (e.g., [33,34,35]). However, at the same time, it essentially does not influence contextual strengthening during suppression phases [34,36]. Instructions to attend to specific letters (targets) would improve dominance of a DRSVP over the other.

The utilization of dichoptic presentations also induces significant alterations in visual processing compared to natural binocular vision conditions or monoptic. Specifically, in our experimental device to deliver dichoptic presentation, the visual axes of the eyes remain parallel, resulting in non-converging gazes. The oculomotor muscles responsible for horizontal eye movements were in a state of rest, and the corresponding neural feedback to the visual system indicates a state of far vergence rather than near vergence [37,38]. On the other hand, the accommodation indicates stimuli very close to the eyes, creating a potential information conflict for the coupling between vergencial movements and accommodation [28]. This conflict of visual information may disturb the binocular visual system in processing stimuli in DRSVP.

Experiment 1 aims to investigate whether the conflict arising from dissimilar information, alongside the disparity and resting vergence state in dichoptic viewing, prolongs the encoding time of the initial target, consequently impairing the detection of the subsequent target.

### 2.1. Method

#### 2.1.1. Participants

24 students (22 females) aged 18–26 years old (M = 22.1; SD = 2.53), with normal or corrected-to-normal visual acuity (20/20) and normal stereoacuity (at least 60 s arc, according to TNO test), right-handed as verified by Edinburgh Handedness Test, from University of Barcelona community, volunteered for the experiment. All participants provided written informed consent. Experiments in this study were approved by the local ethics committee (Bio-ethics committee of the University of Barcelona) and were conducted in accordance with the Declaration of Helsinki of 1975 (as revised in Fortaleza, Brazil, October 2013).

#### 2.1.2. Materials, Stimuli, and Apparatus

**Figure 1:**
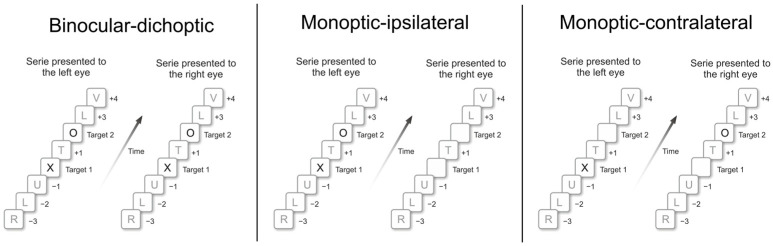
Examples of three types of DRSVP series. (**Left panel**) depicts the DRSVP in binocular-dichoptic condition, in which T1 and T2 (X and O letters) were simultaneously presented to both eyes. (**Central panel**) depicts the monoptic-ipsilateral condition, in which targets were presented only to the left eye. (**Right panel**) depicts monoptic-contralateral condition, in which T1 was presented to left eye and T2, to right eye. In all these examples, T2 was shown in Lag +2, i.e., presented two positions after T1 onset, after a 300 ms temporal interval.

Stimuli were 20 black capital letters (A, C, E, F, H, I, J, K, L, N, O, P, R, S, T, U, V, X, Y, and Z) with 1 cd/m^2^ luminance, approximately 25 mm × 20 mm, inserted in a 40-mm white square background (80 cd/m^2^), subtended a 4° visual angle, including both targets, “X” and “O” letters (Target 1 or T1 and Target 2 or T2). Figure 1 depicts three examples of DRSVP with both targets among some distractor letters. One must note that when the target stimulus (T1 or T2) was presented to only one eye, in monoptic presentations (ipsi- and contralateral), the other eye was shown an empty frame with a uniform white background color.

Stimuli were presented using the Oculus Rift 3D glasses (Development kit 2.0). The Oculus Rift DK 2.0 device, 960 × 1080 pixels spatial resolution for each eye and 120 Hz (60 Hz/eye) frame rate, has two lenses that adapt the image to each eye, covering a 100° field of vision. In this way, we achieve a dichoptic presentation, where stimuli fall on the same retinal coordinates. The Oculus Rift 3D glasses displayed the stimuli to observers’ eyes separately, so that each eye only received one image (dichoptic presentation), that could be the same or different.

A computer with an Intel Core i7 processor and a Nvidia GeForce GTX 1050 graphics card (compatible with OpenGL mode) ran the software program (By Unity Technologies, San Francisco, CA, USA) that generated and presented stimuli, controlled the experimental trials, and recorded the behavioral responses. Observers’ responses, pressing the mouse buttons, were registered by a Unity GL specific software(Uninty. version 2019.4.19f1. San Francisco, CA, USA: Unity Technologies).

#### 2.1.3. Experimental Design

**Figure 2:**
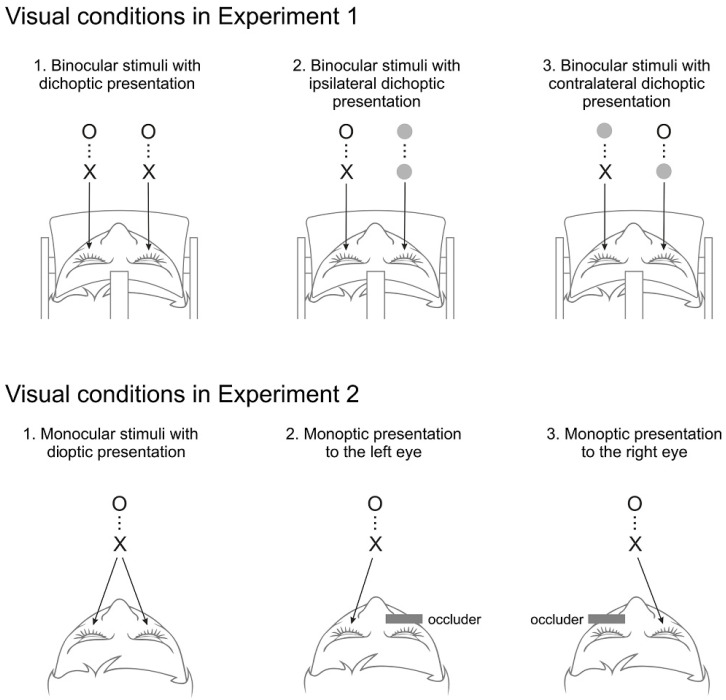
(**Upper panel**): Visual conditions in Experiment 1: Binocular-dichoptic presentation: each eye observed its own DRSVP, both with the two targets; Monocular-ipsilateral presentation: each eye observed its own DRSVP, with only one eye viewing targets; and Monocular-contralateral presentation: each eye observed its own DRSVP, which had either T1 or T2. (**Lower panel**): Visual conditions in Experiment 2: Binocular presentation (monocular stimuli with dioptic presentation), the two eyes observed the single RSVP series, with natural vergencial eye movements; Monoptic presentation to the left eye, with right eye occlusion, only active eye observed the RSVP series; and Monoptic presentation to the right eye, with left eye occlusion, only active eye observed the RSVP series.

The effects of dichoptic presentation on target detection in DRSVP series were investigated in three different conditions of target presentation: (1) a binocular-dichoptic presentation, in which both targets appear to both eyes; (2) a monoptic-ipsilateral, where both targets appear to only one eye (left or right) and empty frame with a uniform white background color were presented to the other eye; and (3) a monoptic-contralateral, where T1 appears to only one eye (left or right) while an empty frame appears to the other eye, and switching eyes on T2 presentation (Figure 2, upper panel).

Each trial consisted of a DRSVP stream of 18 stimuli, which includes either none, one, or two targets among distractors, with a presentation rate of 150 ms per item. Observers responded to four types of DRSVP series differing in the presence or absence of T1 (letter X) and T2 (letter O): (1) one control DRSVP series exhibiting neither T1 nor T2 (no-target); (2) two simple DRSVP series presenting only one target (Only-T1 or Only-T2); and (3) double DRSVP series presenting both targets (T1 + T2). In double DRSVP series, T2 could appear in +1, +2, +3, +4, or +7 positions after T1 presentation position, namely lags +1 to +7. Stimuli were successively presented every 150 ms (6.67 Hz temporal frequency); therefore, the time interval between T1 and T2 was 150 ms for Lag +1; 300 ms for Lag +2; 450 ms for Lag +3; 600 ms for Lag +4; and 1050 ms for Lag +7.

Stimuli randomly appeared within DRSVP series, except for targets T1 and T2. In the Only-T1 series, T1 appeared in either the third, fifth, seventh, or ninth positions and in the Only-T2 RSVP series, T2 appeared between the fourth and 16th positions. In T1 + T2 DRSVP series, T1 also appeared in either the third, fifth, seventh, or ninth positions and T2 appeared in either Lags +1 to +7 (i.e., between the fourth and 16th positions). Observers responded to 54 No-target DRSVP series, 54 Only-T1 DRSVP series, 54 Only-T2 DRSVP series, and 270 T1 + T2 DRSVP series, comprised of 54 series in 5 different Lags. Thus, observers performed 432 trials in total.

Each trial started with a fixation point presented for a variable time around 1 s to the corresponding retinal region for each eye, followed by the DRSVP stream of 18 letters (distractors and targets accordingly to type of DRSVP series). The trial ended with a green question mark, which asked for the observers’ response. In each trial, participants have up to 3 s to make the response after question mark onset. Participants did not receive any feedback. The presentation rate was 6.67 Hz (150 ms/letter), thus each trial took around 5.2 s, considering the fixation (800–1200 ms), DRSVP stream of stimuli, question mark, and response times. The full set of trials lasted about 39–40 min, and each set was divided in three sets of about 13 min, with a 2-min resting time between sets of trials to prevent fatigue.

Participants received the following instructions: “In this task, a very fast sequence of letters will be shown. In some of these sequences, an ‘X’ will appear, and in others it won’t. But, in other sequences, after the ‘X’, an ‘O’ may or may not appear in addition. Also, there is sequences where only an ‘X’ or only an ‘O’ appear. At the end of each sequence, we will ask you to answer as to whether the ‘X’ and/or “O” have appeared or not. If the ‘X’ appeared, you will click the left mouse button, but if after the ‘X’, an ‘O’ also appeared, then you will click the right mouse button. However, if only “O” appeared you will click the central mouse button. Finally, if you did not see an ‘X’ or an “O” at all, don’t click any of the buttons and after 3 s a new trial will start”.

Prior to the experimental tasks, participants underwent a five-trial training period, for practicing the keystrokes associated with the possible responses. Participants responded to the three sets of trials in a single experimental session, with a short interval between each set of trials, when they were allowed to read the instructions again.

#### 2.1.4. Procedures

Each experimental session began with an assessment of the participant’s stereoscopic visual acuity using the TNO Test. Following this, the participant donned the head-mounted display equipped with Oculus 3D glasses, and a stereoscopic pair was presented. The subject was required to report whether it fused into a 3D image. Noticed that, under dichoptic presentations, a calibration was required to adjust the disparity of the presented images in the 3D Oculus glasses to observers’ interpupillary distance, so that they formed images in corresponding points of the two retinas. Then, the participant was instructed in the target detection task in DRSVP, performing five training trials, then the experimental trials began. The participant sat, resting his/her head at a chinrest at 57 cm away from a screen, used solely for the experimenter to follow the trials. The three binocular conditions (binocular-dichoptic, monocular ipsilateral, and monocular contralateral) were randomly presented in three sets of 144 trials (total of 432 trials).

#### 2.1.5. Data Analysis

The main dependent variable was the mean proportion of correct responses for each type of trial: No-target trials, for Only-T1 trials, Only-T2 trials, T1 + T2 trials, separate for each of the five lags in T1 + T2 trials. One must note that in one-target trials (Only-T1 and Only-T2), a correct response is detecting the appearance of one target in DRSVP, while in T1 + T2 trials, observers must correctly detect both targets’ appearance in DRSVP. These variables were submitted to a repeated-measures ANOVA with Greenhouse–Geisser correction for sphericity departures, which was applied when appropriate. Whenever a main effect reached significance, *t*-tests with the Bonferroni correction, to control for the increase in Type I error, verified pairwise comparisons. For significant interactions, the analysis used simple effects test.

In T1 + T2 trials analyses, we used the Only-T1 detection rate as a control performance and compared it to the five lags performances, as usually made in AB research analyses.

### 2.2. Results

#### 2.2.1. Analysis of Control RSVP Series

In the monocular-ipsilateral condition, targets were presented only to the left eye or only to the right eye. In the monocular-contralateral condition, some series presented T1 and T2 to the left and right eye, respectively, while the other series presented in the opposite mapping. In both monocular conditions, the opposing eye to the target presentation viewed an empty frame. As a control for eye dominance effects, we must compare left eye and right eye performances using a paired *t*-test comparing the mean proportion of correct responses in Only-T1 trials. Results showed no reliable differences, *t*(23) = −0.945; *p* = 0.355, between left eye performance in Only-T1 trials (M = 0.718; SEM = 0.040) and right eye (M = 0.764; SEM = 0.036). Therefore, subsequent analyses will collapse both Only-T1 conditions performances.

Following the same procedure, Only-T2 performances from each eye were also compared. Results also showed no reliable differences, *t*(23) = 0.106; *p* = 0.917, between left eye performance in Only-T2 trials (M = 0.697; SEM = 0.030) and right eye (M = 0.692; SEM = 0.035). Therefore, subsequent analyses also will collapse both Only-T2 conditions performances.

**Figure 3:**
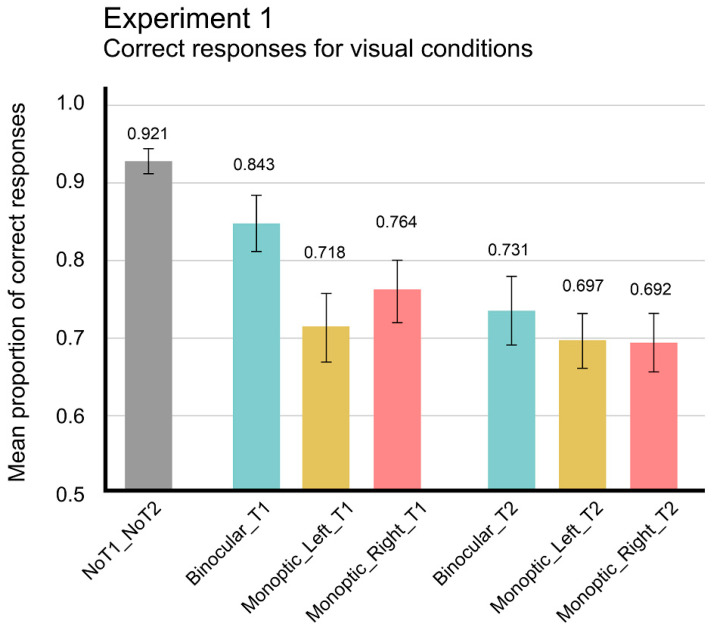
Experiment 1. Average (mean) proportion of correct responses in No-target, ony-T1 and Only-T2 trials as a function of the three visual conditions (Binocular-dichoptic, Monocular-ipsilateral, and Monocular-contralateral) according to the active eye. The error bars indicate standard errors of means (SEM).

Significant differences, *t*(23) = 5.163; *p* < 0.001, were found between No-target (M = 0.921; SEM = 0.015) and Only-T1 performances (M = 0.718; SEM = 0.040) and between No-target and Only-T2 performances (M = 0.697; SEM = 0.030), *t*(23) = 7.601; *p* < 0.001. Figure 3 summarizes the average proportions of correct responses as a function of visual conditions. This may indicate the effect of detecting a target on decision-making accuracy. When observers detected a target, for instance T1, they became primed to expect T2 to appear, and when T2 did not appear, they decreased their confidence on which response to choose. When T1 did not appear, there was no priming effect, lowering the detection rate of second target, which is consistent with poorer performance in Only-T2 binocular trials (M = 0.731; SEM = 0.041) than Only-T1 binocular trials (M = 0.843; SEM = 0.031), *t*(23) = 4.153; *p* < 0.001. However, when T2 was detected, their confidence in their memory of the absence of T1 onset decreased, lowering their confidence on which response to choose even more.

A repeated-measures ANOVA on mean proportion of correct responses with two main factors, two Targets (T1 and T2), and two Visual Conditions (Binocular and Monocular) showed significant differences for Targets, *F*_(1,23)_ = 5.749, *p* = 0.025, *η*^2^*p* = 0.200, *pow.* = 0.632, and Visual Conditions, *F*_(1,23)_ = 8.347, *p* < 0.008, *η*^2^*p* = 0.266, *pow.* = 0.790, and for the interaction Targets × Visual Conditions, *F*_(1,23)_ = 5.327, *p* < 0.030, *η*^2^*p* = 0.188, *pow.* = 0.599. Post hoc analysis, paired *t*-tests with Bonferroni correction, showed reliable differences, *t*_(23)_ = −2.336. *p* < 0.05, in Only-T1 trials between Binocular (M = 0.843; SEM = 0.031) and Monocular (M = 0.718; SEM = 0.040) visual conditions. This indicates that congruent binocular information increased the already higher T1 detection performance.

#### 2.2.2. Analysis of Two-Target DRSVP Series

Analyzing eye dominance effects, a repeated-measures ANOVA on mean proportions of correct responses in T1 + T2 series in Monocular-ipsilateral condition, with two main factors, two Eyes (right and left) and six Lags (0, +1, +2, +3, +4, +7), did not produced a significant effect for main factor Eye, F_(1,3.262)_ = 0.966, *p* = 0.336. Therefore, the following analyses will collapse right and left Eyes series.

For Monocular-contralateral visual condition, a repeated-measures ANOVA on mean proportions of correct responses, with two main factors, two Eyes sequence (left-right and right-left) and six Lags (0, +1, +2, +3, +4, +7), also did not produced significant effects of Eyes sequence. Once again, the following analyses will collapse the two Eyes sequence responses.

**Figure 4:**
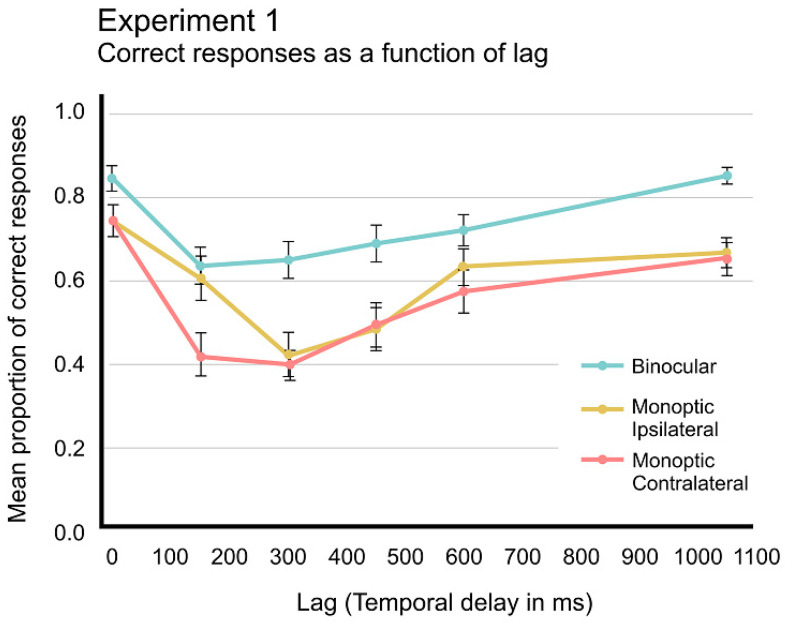
Mean proportion of correct responses as a function of time between T1 onset and T2 onset. Monoptic ipsilateral is depicted by yellow line, Monoptic contralateral, by orange line, and Binocular-dichoptic, by blue line. Note that Lag 0 corresponds to Only-T1 series. The error bars indicate standard errors of means (SEM).

A repeated-measures ANOVA on mean proportion of correct responses, with two main factors, three Visual Conditions (Binocular-dichoptic, Monocular-ipsilateral, and Monocular-contralateral) and six Lags (0, +1, +2, +3, +4, +7), showed significant differences between Visual Conditions, *F*_(1.616,37.167)_ = 46.908, *p* < 0.001, *ε* = 0.808, *η*^2^*p* = 0.671, *pow.* = 1, and Lags, *F*_(2.655,61.057)_ = 25.925, *p* < 0.001, *ε* = 0.531, *η*^2^*p* = 0.530, *pow.* = 1, and also for the interaction Visual Conditions × Lags, *F*_(5.797,133.328)_ = 4.652, *p* < 0.001, *ε* = 0.580, *η*^2^*p* = 0.168, *pow.* = 0.984. Binocular-dichoptic condition (M = 0.729, SEM = 0.028) is reliably different from both Monocular-ipsilateral (M = 0.593, SEM= 0.031, *p* = 0.001) and Monocular-contralateral (M = 0.537, SEM = 0.033, *p* = 0.001) conditions (see Figure 4). Post hoc analysis on reliable interaction also indicated differences in performance between Visual conditions only for Lag +1, where Monocular-ipsilateral performance was equivalent to Binocular condition, although reliably different from Monocular-contralateral, *t*(23) = 5.120; *p* < 0.001.

Binocular-dichoptic condition showed an AB response pattern, although impairment of T2 detection happened earlier in Lag +1, i.e., without Lag 1 sparing, and it took longer than typical AB duration to fully recover T2 detection performance (>600 ms, Figure 4, blue line). In Monocular conditions, both conditions showed an AB response pattern, but only the ipsilateral condition presented the Lag +1 Sparing typical in AB response patterns. One may notice that Monocular conditions presented poorer overall performance than Binocular-dichoptic condition and that both Monocular conditions did not show full recovery of T2 detection performance even after a whole second after T1 onset.

### 2.3. Discussion

Results of Experiment 1 indicated that visual information processed through binocular channels enhances the accuracy of target detection in a dichoptically presented RSVP task. However, it was observed that the AB time course in this visual condition extended beyond the typical range, lasting up to 1000 ms compared to the usual 500–600 ms.

Findings from AB experimental protocols involving amblyopic observers [39] have shown a consistent AB effect without Lag-1 sparing. This response pattern was attributed to either a delay in stimulus processing (as proposed in the eSTST model [24]) or to impaired image quality in the amblyopic eye. Such impairment encompasses deficits in visual acuity and susceptibility to temporal crowding within RSVP, leading to instances of “mis-tokenization”, where a distractor is erroneously bound to the T2 token in the binding pool [39].

The same rationale would extend to dichoptic presentations, which introduce an information conflict at the type analysis stage, thereby prolonging the encoding of T1 [40] and sustaining the reduced reportability of T2 for longer durations than the typical 500 ms duration of the AB effect. Rather than from impaired image quality, as in amblyopic individuals [39], within our experimental framework, this information conflict arises from two primary sources: First, the absence of a perfect match between stimuli presented separately to each eye and their corresponding areas on each retina, and second, disparities in vergence signals from oculomotor muscles when attempting to fuse the images. In the former case, observers’ eyes fail to maintain stable fixation, impeding the establishment of a perfect match between corresponding retinal points receiving stimuli. In the latter case, during dichoptic presentations with binocularly fused stimuli, oculomotor muscles remain in a resting state, signaling far vergence instead of the near vergence state typically associated with stereopsis.

In monocular conditions, where different stimuli images were presented to each eye, increased disparities or reduced contrast were observed, thereby interfering with visual processing. Consequently, this led to a further reduction in the detection rate of the second target, thereby extending the recovery time of the baseline detection rate. This effect was also evident when comparing detection rates in less demanding tasks, such as the control series with only one target, as binocular tasks consistently exhibited higher detection rates compared to monocular conditions (for T1, 0.84 vs. 0.73, and for T2, 0.73 vs. 0.69, respectively).

In the T1 + T2 series, a consistent performance pattern emerges, with the Binocular condition exhibiting higher detection rates than monocular conditions, albeit with a prolonged time course relative to the typical AB effect. However, a noteworthy exception arises. Only the Monocular-ipsilateral condition displayed Lag-1 sparing, a characteristic of the AB effect absent in the Binocular condition. This occurrence may be attributed to the monocular cueing effect [41], which manifests within 150 ms after cue onset and solely within the same eye. When T1 appeared in one eye, it facilitated processing solely in that eye, persisting for 150 ms, thereby leading to Lag-1 sparing. Conversely, this phenomenon did not manifest in Monocular-contralateral conditions. The standard binocular cueing effect needs more time to develop and reaches peak strength after 150 ms [41]. However, within this timeframe, the AB effect is already robust enough to impede facilitation, as the binding pool is engaged in the tokenization process, shutting down attention from the onset of T2.

Following these monocular cueing effects, no further differences were observed between monocular conditions, indicating that this pattern primarily arises from a delay in stimuli analysis (type stage), resulting in errors in the tokenization of potential T2 targets within the binding pool [39]. This effect is exacerbated by the inferior image quality in monoptic presentations, stemming from the visual fusion of the target and an empty frame, which leads to reduced letter acuity. Additionally, susceptibility to temporal crowding in DRSVP, the integration of features across successive letters, further contributes to letter misperception [39].

Our findings suggest that spatial visual attention relies on binocularly tuned information to effectively perform selective attention tasks. When observers processed visual information separately through monocular channels, there was a notable delay in the recovery of target detection rates. Considering the typical patterns of AB responses as indicative of the natural functioning of the BVS in RSVP protocols, it becomes evident that the visual system under dichoptic conditions requires more processing time and exhibits a higher error rate. This delay in processing time can be attributed to information conflicts arising from disparities between the resting state of oculomotor feedback and binocularly fused stimuli, consistent with a near vergence state. Additionally, imperfect spatial matching between stimuli presented to each eye contributes to this delay. It can be argued that dichoptic binocular presentation precludes the natural functioning of the BVS, demanding disengagement of visual attention from the BVS to be directed to monocular channels. Subsequent experiments tested this hypothesis by evaluating performance under more natural conditions, where vergence movements were consistent with binocular information.

## 3. Experiment 2—Monoptic Pressentation

The following experiment was devised to explore whether the conflict arising from divergent vergence movements and binocular information, induced by dichoptic presentations, influenced the performance observed in the previous experiment. Participants viewed a single RSVP displayed on a screen, constituting a monoptic presentation, under three distinct visual conditions: binocular viewing, monocular viewing of the left eye, and monocular viewing of the right eye.

The occurrence of a typical AB effect during monocular viewing of RSVP in this experimental setup would provide evidence supporting the possibility of attentional disengagement from the BVS towards monocular channels. In the previous experiment, dichoptic presentations resulted in prolonged recovery time for target detection, and monocular presentations further exacerbated this effect due to inherent information conflicts, such as incongruence between vergence eye movements and binocular information. Monoptic presentation of a single RSVP, under both binocular and monocular viewing conditions, will yield performances reflecting congruence between vergence movements and binocular information, allowing comparison with performances under dichoptic presentations.

On the contrary, implementing a monocular presentation using an external physical occluder restricts visual processing to occur solely through a monocular channel. This exogenously induced shift of attentional resources to a monocular channel differs fundamentally from the conditions imposed in Experiment 1, where to efficiently detect the target presented to a single monocular channel, observers must have distributed their attention. Eventually, target onset in one monocular channel captured attention to this channel, facilitating second target detection in monocular ipsilateral condition [41].

### 3.1. Method

#### 3.1.1. Participants

Twenty students (18 female), aged between 19 to 24 years old (M = 20.14; SD = 1.96), with normal or corrected-to-normal visual acuity (20/20), normal stereoacuity (assessed by TNO test), right-eye preference and right-handed (assessed by Edinburgh test), from the University of Barcelona community, participated as volunteers in this experiment. All participants gave their written informed consent.

#### 3.1.2. Materials, Stimuli, and Apparatus

**Figure 5:**
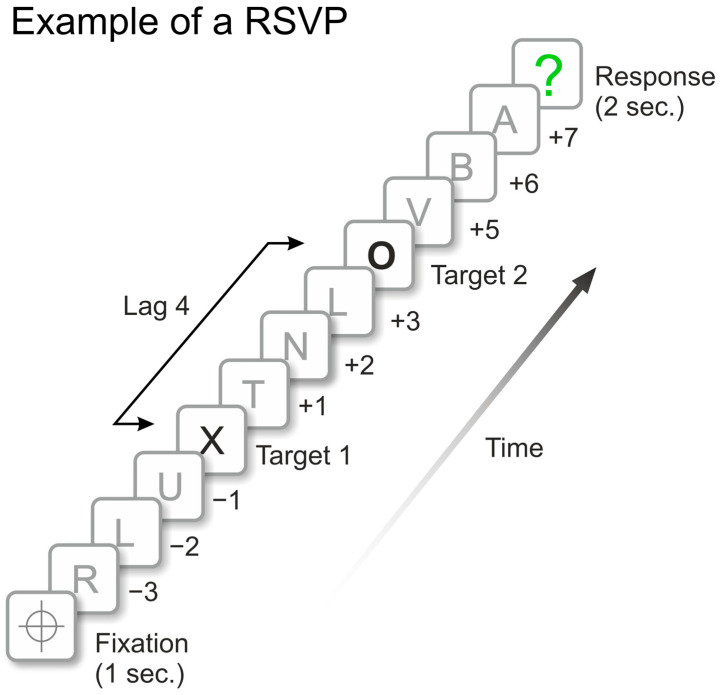
Example of an RSVP series presented on Experiment 2. Stimuli ‘X’ and ‘O’ represent targets T1 and T2, respectively. Remaining letters represent distractors stimuli. The green question mark frame signaled to observers the moment they must make their response. In this example, T2 was showed in Lag +4, i.e., four letters after T1 onset (400 ms temporal interval).

The stimuli consisted of the same black letters from Experiment 1, including the same targets. RSVP series were presented on a 22” LED Samsung flat screen (Samsung Electronics; City: Seoul; and Country Republic of Korea) with spatial resolution of 1920 × 1080 pixels and temporal frame rate 144 Hz, located 57 cm away from the observation point. This viewing distance was guaranteed by a chin rest which held observers’ heads. The black letters (approximately 25 mm × 20 mm) were inserted in 40-mm white circles subtending a 4° visual angle on a black background (1 cd/m^2^). Figure 5 depicts one example of a RSVP with some distractor letters (e.g., “R” and “L”, etc.), targets T1 and T2, and the green question mark that asked for the observers’ response.

The same computational equipment from Experiment 1 was used in this Experiment, except the use of Oculus 3D glasses for dichoptic presentation. Observers’ responses, pressing the mouse buttons, were registered by Unity GL specific software, as in Experiment 1.

#### 3.1.3. Experimental Design

Observers viewed a single RSVP series on the screen either simultaneously with the two eyes or with only one unoccluded eye (either the left or right eye). In this last condition, the other eye was occluded by a patch (Figure 2 lower panel). Under these three visual conditions, participants directly viewed the screen with a binocular viewing condition prior to the monocular conditions.

Each trial consisted of a stream of eighteen stimuli, which includes either none, one, or two targets among distractors, with a presentation rate of 100 ms per item, followed by a green question mark frame, which asked for observer’s response. Observers responded to four types of RSVP series: (1) No-target RSVP series, without any target; (2) two simple RSVP series with only one target (Only-T1 or Only-T2); and (3) double RSVP series with both targets (T1 + T2). Stimuli randomly appeared within DRSVP series, except for targets T1 and T2. In the Only-T1 series, T1 appeared in either the third, fifth, seventh, or ninth positions and in the Only-T2 RSVP series, T2 appeared between the fourth and fifteenth positions. In T1 + T2 series, T2 could appeared in Lags +1, +2, +3, +4, or +6 positions after T1 onset. Since stimuli were presented every 100 ms, the time interval between T1 and T2 was 100 ms for Lag +1; 200 ms for Lag +2; 300 ms for Lag +3; 400 ms for Lag +4; and 600 ms for Lag +6. In each viewing condition, observers responded to 12 No-target trials, 12 Only-T1 trials, 12 Only-T2 trials, and 60 T1 + T2 trials, considering 12 trials for each of the 5 Lags. Thus, observers performed 96 trials for each visual condition, a total of 288 trials for the entire experimental session.

#### 3.1.4. Procedures

Participants sat in front of the screen, resting their head in the chinrest located 57 cm away from the screen. The experimental session began with TNO Test assessing the stereoscopic visual acuity. Participant listened to the same instructions as Experiment 1 and performed five training trials, and then the test began. The test followed this order: first, binocular viewing condition; second, monocular viewing with occlusion of the non-preferred eye (left eye, since all observers have right-eye preference); and finally, monocular viewing with occlusion of the preferred eye. A brief resting time (around two minutes) was allowed between these conditions. Each viewing condition session lasted about seven minutes, then the whole experimental session lasted approximately 30 min. Participants responded to the three set of trials in a single experimental session, with a short interval between each set of trials when they were allowed to read the instructions again.

#### 3.1.5. Data Analysis

The main dependent variable was the mean proportion of correct responses for each. In this case, the italic letters facilitate the distinction between the factors analyzed.type of trial, which were submitted to repeated-measures ANOVAs with Greenhouse–Geisser correction for sphericity departures. Whenever a main effect reached significance, pairwise comparisons were performed by *t*-tests with Bonferroni correction to prevent the likelihood of Type-I error. For significant interactions, the analysis used simple effects test. In T1 + T2 trials analyses, performance on Only-T1 trials served as baseline, as usually made in AB experimental protocols.

### 3.2. Results

#### 3.2.1. Analysis of Control RSVP Series

**Figure 6:**
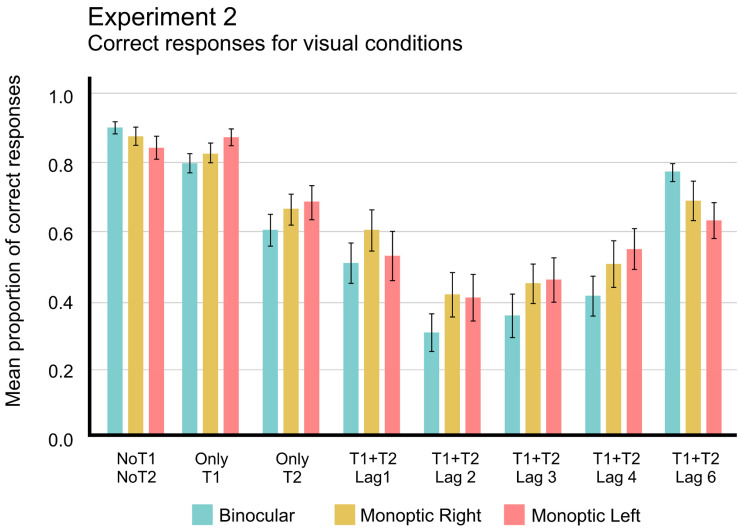
Mean proportion of correct responses for each viewing condition and type of trials. Error bars indicate SEM.

A repeated-measures ANOVA (with Greenhouse–Geisser correction when appropriate) on mean proportion of correct responses, with two main factors, three Viewing conditions (Binocular, Monocular preferred eye, or Monocular non-preferred eye) × two Targets (T1 or T2) showed significant differences for both main factors: Viewing conditions, *F*_(1.590,30.201)_ = 3.527, *p* < 0.039, *η*^2^*p* = 0.157, *pow.* = 0.551, and Targets, *F*_(1,19)_ = 18.133, *p* < 0.001, *η*^2^*p* = 0.488, *pow.* = 0.981. Nevertheless, no reliable differences were found for the interaction between these factors, *F*_(1.856,35.259)_ = 0.201, *p* = 0.819), nor for post-hoc analyses comparing the viewing conditions effects (*p* = 0.120). Figure 6 summarizes the mean proportion of correct responses.

Only-T2 detection rate (M = 0.653, SEM = 0.040) was worse than Only-T1 performance (M = 0.835, SEM = 0.021), supporting the same hypothesis from Exp. 1. Detecting the first target activated a priming to T2 appearance, but in Only-T1, when T2 did not appear, it decreased their confidence on which response to choose. In Only-T2, when T1 did not appear, there is no priming effect, lowering the detection rate of second target. This effect is combined to a decrease in confidence on response associated to a lowering of confidence in the memory of the absence of T1 onset after T2 detection. These effects are consistent with poorer performance in overall Only-T2 than overall Only-T1.

#### 3.2.2. Analysis of Two-Target RSVP Series

**Figure 7:**
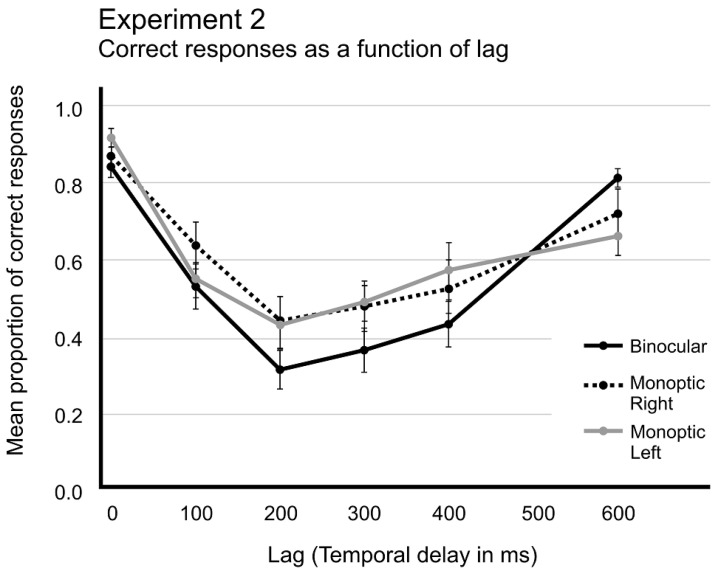
Mean proportion of correct responses as a function of lag, comparing the three visual conditions, binocular, monocular right eye, and monocular left eye. The error bars indicate standard errors of means (SEM).

A repeated-measures ANOVA (with Greenhouse–Geisser correction when appropriate) on mean proportion of correct responses, with two main factors, three Viewing conditions (Binocular, Monocular right eye, and Monocular left eye) × six Lags (0, +1, +2, +3, +4, +6), showed reliable differences for Lag, *F*_(2.663,50.591)_ = 25.278, *p* < 0.001, *η*^2^*p* = 0.571, *pow.* = 1.0, and also for the interaction Viewing conditions × Lag, *F*_(5.065,96.237)_ = 3.109, *p* < 0.012, *η*^2^*p* = 0.141, *pow.* = 0.861. However, Visual Conditions did not produce significant differences, *F*_(1.763,33.502)_ = 2.947, *p* < 0.072, *η*^2^*p* = 0.134, *pow.* = 0.504. Figure 7 shows these results.

Post hoc analyses revealed that the mean proportion of correct responses in Lag +1, Lag +2, Lag +3, and Lag +4 reliably differed from Only-T1 performance for the three visual conditions. The exception was the Lag +6 in the binocular condition, which did not differ from Only-T1 detection rate, *t*_(19)_ = −0.827, *p* = 0.419.

### 3.3. Discussion

Experiment 2 aimed to investigate the attentional blink under monoptic visual conditions to provide comparisons with the dichoptic presentations from Experiment 1. Unlike Experiment 1, all three visual conditions in Experiment 2 exhibited the typical AB pattern, characterized by Lag-1 sparing and the lowest detection rate occurring at Lag +2 (after 200 ms), followed by a progressive recovery of the detection rate up to Lag +6 (after 600 ms). Although the monocular conditions in Experiment 2 did not fully recover the detection rate at Lag +6, the presence of the same parabolic curve in detection performance suggests that full recovery will likely occur shortly after Lag +6.

The results revealed the occurrence of the AB effect under monocular viewing conditions with monoptic presentations, indicating the capacity for directing attention to monocular channels of visual processing. Since accommodative vergence and fusional vergence information remain consistent under monoptic viewing conditions [28], there was no conflicting information causing a delay in the tokenization stage in Experiment 2. Thus, the typical AB response patterns depend on the natural functioning of the BVS in RSVP protocols, which was hindered in Experiment 1, where vergence movements were inconsistent with binocular information. It can be argued that monoptic presentations facilitated the natural functioning of the BVS, even in monocular conditions, by providing consistent accommodative vergence movements [42], despite the absence of binocularly fused images. Consequently, visual attention could be effectively directed to monocular channels, despite the lower image acuity in monocular conditions. The diminished image acuity in monocular presentations resulted in a minor delay in the tokenization process [40], contributing to the delay in the recovery of the detection rate of T2.

**Figure 8:**
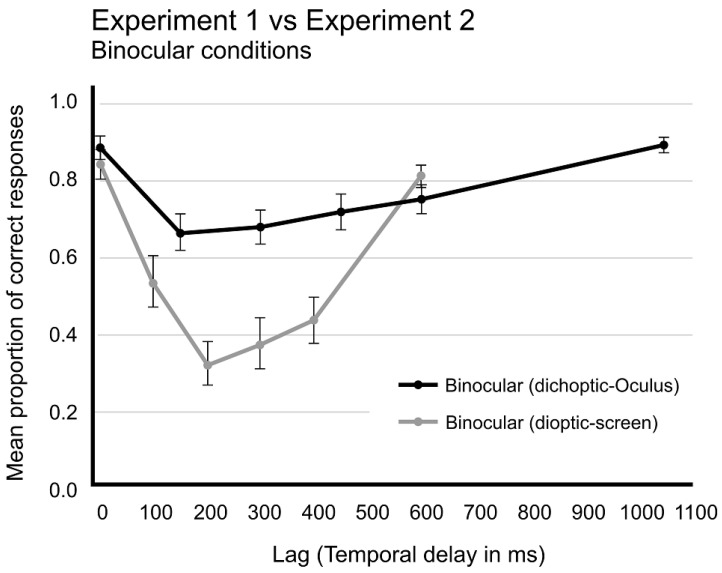
Comparison of proportion of correct responses as a function of time in binocular conditions from Experiments 1 and 2. Black line represents Dichoptic presentation from Experiment 1 and grey line, Dioptic presentation from Experiment 2. Vertical bars represent SEM.

However, when comparing the results from both Experiments 1 and 2, different perspectives on this interpretation may arise. Figure 8 illustrates the performances in the two binocular conditions from Experiments 1 (dichoptic, using the Oculus apparatus) and Experiment 2 (monoptic, with a single RSVP presented on the screen). A notable difference in performances is observed. These distinctions can be explained by two hypotheses. One hypothesis suggests that dichoptic presentation resulted in a dissociation of the monocular visual channels, imposing separate processing of these channels. Since the visual system did not receive any convergence signal from the oculomotor systems, a conflict arose due to the absence of expected disparity in images from both eyes. This cue conflict in binocular fusion processing led to a processing delay, impacting the AB mechanism and extending the tokenization process up to 1000 milliseconds, thereby prolonging the time required to recover T2 detection rates beyond the usual AB time course.

The second hypothesis proposes that attention operates as a late filter, acting after the binocular fusion of images captured by both eyes. Consequently, prior to fusion, monocular images do not receive attention; that is, attentional resources cannot be divided between monocular channels. Therefore, the AB occurred only in the monoptic condition, while a distinct attentional phenomenon manifested in dichoptic presentations. This explanation could elucidate the disparity in performances observed between Experiment 1, where the binocular condition significantly outperformed the monocular ones, and Experiment 2, where performances under both binocular and monocular conditions were nearly equivalent.

**Figure 9:**
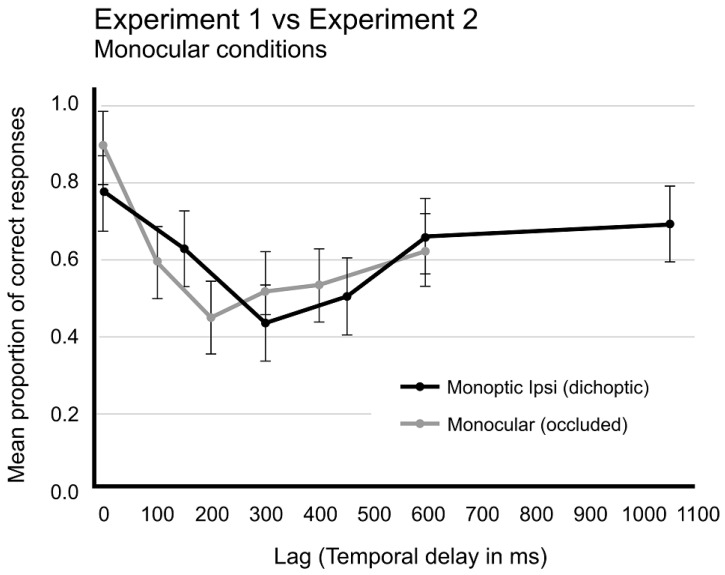
Comparison of proportion of correct responses as a function of time in Dichoptic Monocular-ipsilateral condition from Experiment 1 (black line) and collapsed Monoptic Monocular conditions from Experiment 2 (grey line). Vertical bars represent SEM.

When comparing Dichoptic Monocular-ipsilateral, which exclusively presented targets to one eye, with Monoptic Monocular presentations (Figure 9), where the other eye was covered, two hypotheses may be considered. One hypothesis posits that dichoptic presentation of targets implicitly captured attention to a single monocular channel, since attention initially are evenly distributed between eyes. T1 onset captured attention to its monocular channel facilitating detection of T2 onset in ipsilateral condition, but impairing T2 detection in contralateral conditions. In contrast, in monoptic conditions, since one eye is covered, attention was forcedly concentrated on the other eye. These differences in attentional allocation could underlie the observed performance disparities.

Alternatively, these performance differences could stem from binocular competition occurring in Dichoptic Monocular presentations from Experiment 1, which was absent in Experiment 2 where observers viewed stimuli through only one eye. Therefore, in the Monocular conditions of Experiment 1, where dissimilar stimuli were presented to both eyes at target positions, the visual conflict of binocular competition might have prolonged the tokenization process, resulting in an extended impairment in T2 detection.

However, a third alternative, arising from the combination of the two previous hypotheses, suggests that the performance pattern resembling a binocular rivalry effect could be solely attributed to the alternation of attention between the two eyes. In essence, the attentional switching between eyes in the dichoptic condition may replicate the effects of binocular rivalry in target detection during the RSVP task. This hypothesis is supported by the performance in the monocular-contralateral condition in Experiment 1, which required attentional shifts between eyes and resulted in a greater delay in target processing time, thus preventing Lag-1 sparing. This delay could be attributed to a monocular cueing effect [41] rather than a potential binocular rivalry effect.

## 4. Experiment 3—Binocular Competition in DRSVP Presentations

Experiment 3 addressed the last hypothesis stated in Experiment 2 discussion, i.e., that alternating attention between eyes would mimic the effects of binocular rivalry in DRSVP tasks. The dissimilarity of the target images could, under certain exposure times, promote binocular rivalry effects [43]. However, in our Dichotic RSVP tasks, exposure times of hundreds of milliseconds may be not sufficient to generate the perceptual alternation of binocular rivalry, which fluctuates irregularly over time, with a competing perception dominating the other for several seconds [13]. Therefore, target presentation occurred during the amount of time of a dominant phase of one of the competing stimuli, while the other is suppressed.

In an experimental setting where both targets are presented simultaneously, but separately to each eye, if given sufficient exposure time, binocular rivalry would occur and both targets should be eventually detected, for there would be a perceptual alternation between target perceptions. Since our DRSVP tasks allowed insufficient time for perceptual alternation to occur, we predict that observers will present a lower detection rate of targets in Rival conditions (for one or two targets) when compared to binocular presentation.

Instead of binocular rivalry, dichotic presentation of a target simultaneously to another target or to a distractor would create a binocular competition. Since observers’ responses were dependent on correctly perceiving targets, whether there appeared a single or two targets, performance would diminish accordingly to uncertainty caused by binocular competition.

### 4.1. Method

#### 4.1.1. Participants

Eighteen students (16 females and 2 males) aged 18–26 years old (M = 22.1; SD = 2.53), with normal or corrected-to-normal visual acuity (20/20) and normal stereoacuity (at least 60 s arc, according to TNO test), all with right-eye preference and right-handed as determined by Edinburgh test, from University of Barcelona community, volunteered for the experiment. All participants provided written informed consent. Experiments in this study were approved by the local ethics committee.

#### 4.1.2. Materials, Stimuli, and Apparatus

The same computational equipment from Experiment 1 ran the software program (By Unity Technologies) that generated and presented stimuli, controlled the experimental trials, and recorded the behavioral responses. Observers’ responses, pressing the mouse buttons, were registered on Unity GL specific software. The stimuli consisted of the same 18 black capital letters in Experiment 1, presented dichoptically using the Oculus Rift (Development kit 2.0) 3D glasses. 

**Figure 10:**
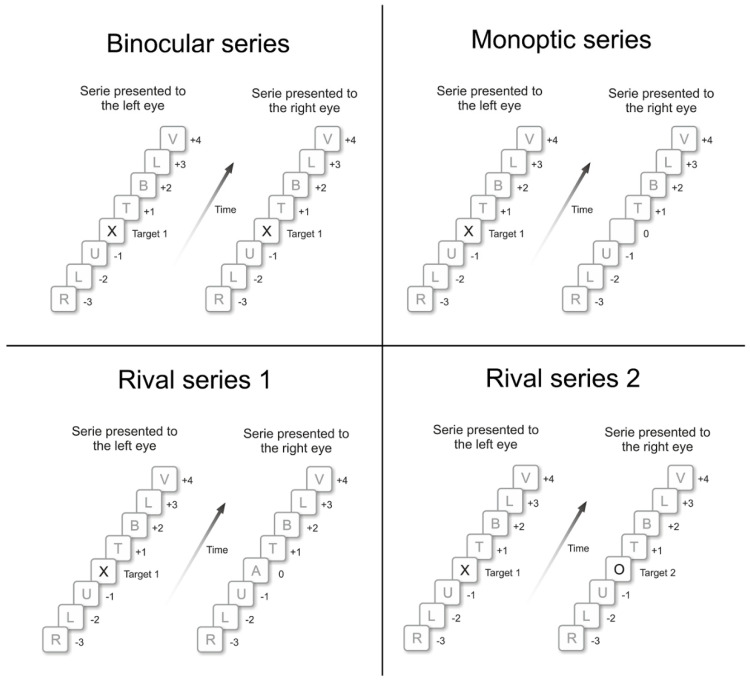
Example of four experimental dichoptic conditions presented on Experiment 3. Letters X and O represent targets T1 and T2, respectively, while remaining letters were distractors. (**Binocular Series**): Binocular condition, targets were simultaneously presented to both eyes. (**Monoptic Series**): Monoptic presentation, in which T1 (X letter, in this case) was presented to only one eye, in the example, to the left eye, and an empty frame to the other eye. (**Rival Series 1)**: Rival 1 condition, a target (“X”, in this case) was presented to one eye and a distractor (“A”, in this case) to the other eye. (**Rival Series 2**): Rival 2 condition, a target (“X”, in this case) was presented to one eye and another target (“O”, in this case), to the other eye.

Figure 10 depicts four examples of RSVP series with targets (T1, the letter “X”; and T2, the letter “O”) and distractors (e.g., letters “R” and “L”). Every RSVP series ended with a green question mark, which asked for the observers’ response. One must note that, in monocular condition, when the target was presented to only one eye, an empty frame with a uniform white background color was presented to the other eye (Panel B).

#### 4.1.3. Experimental Design

Four different dichotic presentations investigated whether binocular rivalry affected observers’ performance in our experimental setting. In Binocular presentation, the same target (T1 or T2) was presented to both eyes. This was a control condition where congruent stimuli would not produce any binocular rivalry. In Monocular presentation, a target (T1 or T2) was presented to only one eye, while an empty frame (blank frame) was presented to the other eye. These conditions verified whether the absence of a rival stimulus in the opposite eye would elicit a binocular rivalry effect, since present evidence is controversial in this regard [13,14,15,16].

In Rival 1, a target (T1 or T2) was presented to only one eye, while a distractor was presented to the other eye. In Rival 2, T1 and T2 were simultaneously presented to different eyes. Both conditions are aiming at determining whether binocular rivalry would play a role in detection performances in our experimental settings, where Rival 2 condition verified whether target detection increased binocular rivalry effects.

#### 4.1.4. Procedures

Participants sat in front of the screen, resting their head in the chinrest located 57 cm away from the screen. The experimental session began with TNO Test assessing the stereoscopic visual acuity. Prior to the experimental tasks, participants underwent a five-trial training period to practice the keystrokes of responses.

The test session presented five types of DRSVP in random order: 24 No-Target series as a control; 24 Only-T1 series, in which T1 (X) was presented to only one eye, while an empty frame was presented to the other eye; 24 Only-T2, in which T2 (O) was presented to only one eye, while an empty frame was presented to the other eye; 24 Rival-1 series, in which one target (T1 or T2) was presented to one eye, while a distractor (e.g., A) was simultaneously presented to the other eye; and 24 Rival-2 series, in which each target was simultaneously presented to one eye. Thus, a test session comprised 120 trials.

As in Experiment 1, each trial consisted of a DRSVP stream of 18 stimuli, starting with a fixation point, which lasted 800–1200 ms, followed by a DRSVP, then a green question mark asked for observers’ response, with a time window expiring 3 s after stimuli offset, then another trial begin, without feedback about response. The presentation rate was 6.67 Hz (150 ms/letter); thus, a single trial took around 5.2 s, considering the fixation (800–1200 ms), DRSVP series, question mark, and response times. Test sessions were divided into three series of trials of approximately 12 min, thus lasting approximately 36 min, with a brief resting time (around 2 min) between these series of trials.

Note that in the present experiment, there were no lags, since targets were presented either isolated or simultaneously, and not sequentially. Targets could appear in either third, fifth, seventh, or ninth positions in DRVSP series.

Participants received the following instructions: “In this task, a very fast sequence of letters will be shown. In some of these sequences, an ‘X’ will appear, and in others, it won’t. In some sequences, an ‘O’ may appear in addition. Also, there is sequences where only an ‘X’ or only an ‘O’ will appear. At the end of each sequence, we ask you to respond as to whether the ‘X’ and/or ‘O’ have appeared or not. If the ‘X’ appeared, you will click the left mouse button, and if both ‘X’ and ‘O’ appeared, then you will click the right mouse button. However, if only “O” appeared, you will click the central mouse button. Finally, if you did not see an ‘X’ or an ‘O’ at all, don’t click any button and, after 3 s, a new trial will start”. Participants responded to the full set of trials in a single experimental session, with a short interval between each set of trials, when they were allowed to read the instructions again.

#### 4.1.5. Data Analysis

The main dependent variable was the mean proportion of correct responses for each type of trial, which were submitted to repeated-measures ANOVAs with Greenhouse–Geisser correction for sphericity departures. Whenever a main effect reached significance, pairwise comparisons were performed by *t*-tests with Bonferroni correction to prevent the likelihood of Type-I error. For significant interactions, the analysis used simple effects test.

### 4.2. Results

For each participant, we computed the mean proportion of correct responses in the four conditions, Binocular, Monocular, Rival 1, and Rival 2, for Target 1 and Target 2, for four positions (3rd, 5th, 7th, or 9th), and for active eye (left or right eye). To verify the possibility of binocular rivalry in our experimental setting, a one-way ANOVA with Visual Conditions as within-subjects factor (Binocular, Monocular, Rival 1, or Rival 2) was run on mean individual proportion of correct responses collapsing both targets (T1 and T2), eyes (left and right), and target positions (3rd to 9th), with Bonferroni correction for the multiple comparisons. The analyses revealed reliable differences between Visual Conditions, *F*_(3,140)_ = 25.939, *p* < 0.001. Post hoc analyses showed significant differences between the pairs, Binocular (M = 0.796; SEM = 0.023) and Rival 1 conditions (M = 0.521; SEM = 0.028), *p* < 0.002; Binocular and Rival 2 (M = 0.387; SEM = 0.058), *p* < 0.001; Monocular (M = 0.712; SEM = 0.024) and Rival 1, *p* < 0.001; and Monocular and Rival 2, *p* < 0.001. No significant differences were found between Rival conditions, nor between Binocular and Monocular conditions.

**Figure 11:**
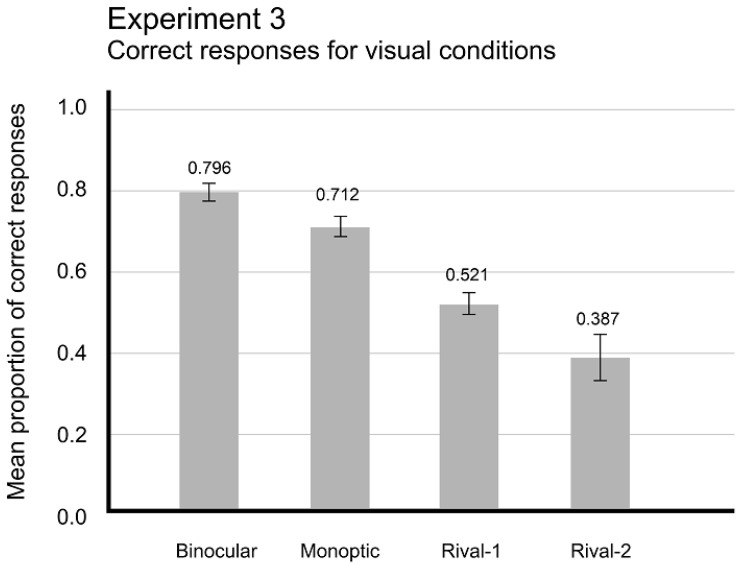
Mean proportion of correct responses for each visual conditions, collapsing targets (T1 and/or T2), serial position (3rd, 5th, 7th, and 9th) and eyes (right and left). Error bars indicate SEM.

Figure 11 shows the mean proportions of correct responses for each visual condition. One may notice that visual conditions of binocular competition, Rival 1 and 2 conditions (M = 0.521 and M = 0.387, respectively), detection performance was significantly lower than in visual conditions without competition between stimuli (Binocular, 0.796, and Monocular, 0.712). Monocular condition (M = 0.712) had performance comparable to Monocular Only-T1 and Only-T2 trials from Exp. 1 (M = 0.718 and M = 0.697, respectively). Binocular condition, which collapsed performances to both targets, T1 and T2, presented a performance (M = 0.796) consistent to Binocular Only-T1 and Only-T2 conditions from Exp. 1 (M = 0.843 and M = 0.731, respectively).

**Figure 12:**
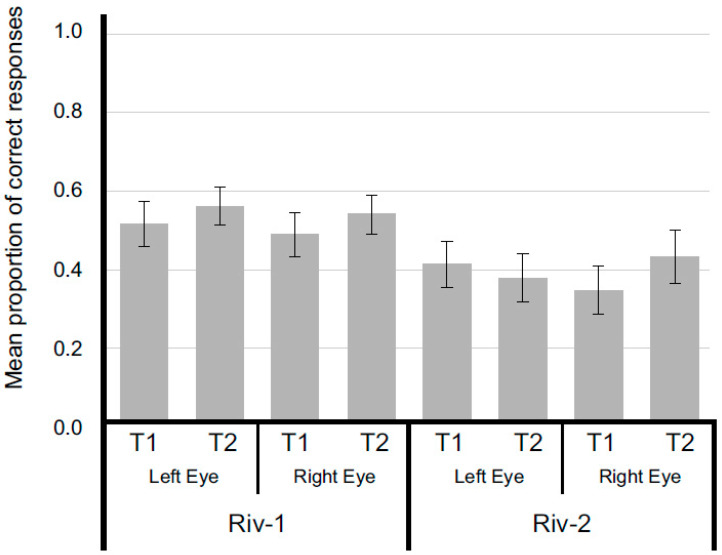
Mean proportion of correct responses as a function of experimental conditions, targets (T1 or T2), eye (left and right eyes), and competing stimulus (distractor or other target) of Rival conditions of Experiment 3. Error bars represent SEM.

Aiming to investigate the differential effects of the binocular competition produced either by a distractor or by another target, we calculated the mean proportion of correct responses for each participant and ran a repeated-measures ANOVA with three within-subjects factors, Rival letters (distractor or target), Targets (T1 or T2), and Eyes (left or right eye), which produced reliable differences only for main effect, Rival letter, *F*_(1,35)_ = 7.048, *p* = 0.012, *η*^2^*p* = 0.168, *pow* = 0.733, which is summarized in Figure 12. Significant differences between Rival 1 and Rival 2 may be due to the differential effect of the requirements for observers to choose a correct response in both conditions. In Rival 1, a correct response is dependent on the correct detection of only a single target, which is competing with a distractor. The likelihood of selecting response for perceiving only one target increased when one target is detected, while the likelihood of detecting both targets is severely impaired by binocular competition between stimuli. The binocular competition between stimuli produced a masking effect due to simultaneous presentation of target and distractor or both targets. The masking effect is responsible for the reduced accuracy in observers’ performance (M = 0.521; SEM = 0.028) when compared to Monocular condition (M = 0.712; SEM = 0.024), where only an attenuation of target would be occurring due to simultaneous presentation of target and an empty frame.

In Rival 2, a correct response is dependent on the correct detection of both targets, which are competing due to simultaneous presentation. Therefore, due to the masking effect of binocular competition between left and right stimuli, the diminished accuracy is directly related to an even more restricted probability of correct detection of stimuli. Since the only correct response among the four possible responses would be detecting both targets being presented simultaneously, and the likelihood of detecting both is severely diminished by binocular competition, observers presented an even lower detection rate (M = 0.387; SEM = 0.058) compared to Rival 1 (M = 0.521; SEM = 0.028).

### 4.3. Discussion

Experiment 3 was conducted to explore whether binocular competition between stimuli influenced the performance pattern observed in monocular conditions during dichoptic presentation. In the Rival conditions, where two stimuli (either a distractor or another target) were simultaneously presented, lower detection rates were observed compared to monocular conditions. This indicates that the detrimental effect on detection rates caused by binocular target competition was more pronounced than that of a single monocularly processed target. Consequently, it can be inferred that binocular competition, which was absent in the monocular condition due to simultaneous presentation of targets with an empty frame, did not account for the performance observed under monocular dichoptic presentations.

An alternative explanation could be the inability to direct attention to monocular visual processing channels, as this attentional orientation occurs in stages subsequent to binocular fusion. In other words, the attentional control process would only occur after the binocular fusion stages of visual processing in the RSVP. In dichoptic RSVP presentations, slight inaccuracies in the fusional mapping of binocular information and conflicts between oculomotor feedback and accommodative vergence information could slow down the tokenization process, assumed in the eSTST model [24]. In monocular dichoptic presentations, the target presented to only one eye introduces an additional effect on binocular fusion bias, resulting in a kind of attenuation of the target. This attenuation makes the type analysis process and completion of the tokenization stage even more challenging [40].

## 5. General Discussion

The primary objective of this set of experiments was to investigate whether it is possible to direct attention to monocular channels using AB effect protocols, which involve conscious attention processes. In Experiment 1, we aimed to determine whether observers could allocate their attention to monocular channels of visual processing in AB effect protocols by separating monocular channels in dichoptic presentations and presenting targets to either one or both eyes. We found that dichoptic presentations resulted in a delay in the type stage of the eSTST model [24], postponing the subsequent tokenization process, as predicted elsewhere [40]. This led to an elongation of the detection rate curves, delaying the recovery of detection rates until one full second after the onset of T1. The delay in monocular conditions may be further explained by a masking effect or visual attenuation, possibly due to the target being visually fused with a simultaneous empty frame [39].

In Experiment 2, we utilized monoptic presentations to circumvent the conflict between accommodative vergence and feedback from the oculomotor system that occurred in dichoptic presentations. We aimed to determine whether observers could deliberately direct their attention to monocular channels when compelled to do so. Partially occluding one eye resulted in a slight yet significant increase in the recovery time of detection rates, while preserving the curve shape and other features (e.g., Lag-1 sparing) comparable to those observed in binocular conditions. These findings suggest that observers effectively directed their attention to monocular channels when such focus was externally imposed by occluding the other eye. A more implicit, endogenous distribution of attention, as observed in the dichoptic monocular task, adversely affected performance. While observers demonstrated the ability to allocate attention to a monocular channel, this capability was only evident in conditions where there was no visual conflict between vergence and binocular cues, such as those encountered in the monoptic conditions of Experiment 2.

In Experiment 3, we solely investigated the potential influence of binocular competition within our dichoptic experimental settings. Our findings indicated that binocular competition did not account for monocular performance in dichoptic presentations, as evidenced by the significant differences observed between the monocular conditions of Experiment 1 and the rivalry conditions of Experiment 3. In the Rival conditions, where stimuli were simultaneously presented to each eye, detection rates significantly decreased compared to the monocular conditions of Experiment 1. The differential impact of contrast attenuation caused by empty frame presentation and binocular competition was evaluated by comparing the monocular and Rival conditions of Experiment 3. This comparison revealed the adverse effect of competing stimuli on detection rates. Notably, while the monocular conditions in both Experiments 1 and 3 demonstrated similar performance levels, the Rival conditions in Experiment 3 exhibited significantly lower detection rates.

The present study has several limitations. First, differences in the concepts of eye preference and visual field dominance [30] suggest the possibility of a visual field dominance effect on our results. However, the lack of significant effects of the eye of target presentation in most experimental conditions may mitigate the relevance of this limitation. Second, the differential effect of chosen letters as targets should have biased results, since X may be more easily detected than O. Future research should address this issue.

Third, presenting target with an empty frame decreased its visual contrast, then turning it as perceptually salient, i.e., target would stand out from the remaining stimuli, which have an almost constant contrast. The salience of T2 salience has a larger effect than T1 salience, almost eliminating the AB [44]. Future studies should consider a control condition for contrast reduction, without contrast change, in the same experimental settings of monocular condition of Experiment 2, to verify whether AB pattern changes with the same time course as occurred in Experiment 1.

Another limitation is associated with the use of an occluder in Experiment 2, which compelled the allocation of attentional resources to a single eye. Experiment 2 did not address whether attentional distribution could be voluntarily directed to a single eye when both eyes were simultaneously assessing the RSVP.

## 6. Conclusions

The AB effect is a phenomenon that provides insights into visual awareness, specifically how the visual system constructs the succession of perceived visuospatial events in conscious experience. In RSVP series, visual information is rapidly forgotten unless working memory is recruited for its processing [22]. This processing relies on the attentional enhancement mechanism hypothesized in the eSTST model [24], known as the blaster, which may be triggered by top-down processes, such as the provided experimental instructions. The blaster mechanism generates a brief period of attentional enhancement for stimuli, when visual representations transition into tokens in working memory [24,25]. Consequently, once the blaster is activated, the likelihood of visual awareness of stimuli increases.

Attentional capture, whether induced by top-down processes or exogenous cueing, heightens the likelihood of initiating the tokenization stage, thereby inhibiting the blaster and resulting in the AB phenomenon. In essence, the consolidation of the episode (token) in working memory impedes attentional deployment. This effect was more pronounced under conditions of increased T1 encoding difficulty, such as in dichoptic conditions, due to cue conflict between vergence movements and binocular information. One factor known to attenuate the AB effect is precueing T2 [40], as observed in the monocular ipsilateral condition of Experiment 1, where the monocular cueing effect [41] led to differences in the occurrence of Lag-1 sparing.

In summary, this study underscores the intricate relationship between visual attention and binocular vision, shedding light on how these processes interact during tasks, such as RSVP. It highlights notable differences between dichoptic and monoptic presentations of stimuli, particularly in the context of the AB effect, which appears to be attenuated or absent in dichoptic settings. Our findings suggest that attention cannot be distributed between monocular channels in an implicit, endogenous manner. Only when attention is forced through a single monocular channel, such as when one eye is occluded, and also under conditions where there is no conflict between oculomotor signals and vergence movements, can attention be shifted to a monocular channel with minimal loss in the recovery time of detection rates. Thus, the visual awareness of discrete, rapid, and successive events depends on the consistency of binocular and vergence cues and may not be implicitly directed to a monocular channel. Future research should investigate whether attention can be voluntarily directed to monocular channels under binocular viewing conditions to achieve a more comprehensive understanding of the processing chain involved in the conscious perception of individual stimuli among multiple stimuli.

## Data Availability

The datasets analyzed during the current study for all experiments are available at OSF Data repository: https://osf.io/8cqgu/?view_only=de5a6f90d8364f16b7ce28d184f6f09a, accessed on 16 November 2011.
